# The effect of healthy life behaviors in pregnancy on pregnant sleep quality

**DOI:** 10.1590/1806-9282.20241821

**Published:** 2025-10-17

**Authors:** Nazan Karahan, Sümeyra Damsarsan, Şerife İrem Döner, İlknur Sirkeci

**Affiliations:** 1 University of Health Sciences, Gülhane Faculty of Health Sciences, Department of Midwifery – Ankara, Turkey.; 2 Ankara Medipol University, Faculty of Health Sciences, Department of Midwifery – Ankara, Turkey.; 3 Etlik Zübeyde Hanım Gynecology and Pediatrics Training and Research Hospital – Ankara, Turkey.

**Keywords:** Sleep quality, Pregnancy, Health behavior, Prenatal care, Sleep

## Abstract

**OBJECTIVE:**

The relationship between sleep quality in pregnancy, which is associated with adverse pregnancy outcomes, and healthy life behaviors is unclear. The aim of the study was to investigate the relationship between healthy life behaviors and sleep quality during pregnancy.

**METHODS:**

This study was conducted as a descriptive and correlational study. This study included 282 pregnant women. The study "Healthy Lifestyle Behaviors in Pregnancy Scale" was used to determine women's healthy lifestyle behaviors and "Pittsburgh Sleep Quality Index" was used to evaluate sleep problems. In this study, PATH analysis was used to test the model (p<0.050).

**RESULTS:**

The mean age of the participants was 27.43±5.54 years, and the mean gestational age was 24.32±10.4 weeks. Healthy Lifestyle Behaviors in Pregnancy Scale total mean score was 109.62±16.46, and Pittsburgh Sleep Quality Index total mean score was 6.90±3.37. According to the results of path analysis, there was no statistically significant relationship between Pittsburgh Sleep Quality Index and Healthy Lifestyle Behaviors in Pregnancy Scale total and sub-dimension scores (p>0.050).

**CONCLUSION:**

The results of this study suggest that there is no association between healthy lifestyle behaviors and sleep quality during pregnancy, but poor sleep quality is common during pregnancy. Therefore, studies investigating the longitudinal associations of sleep with objectively measured lifestyle behaviors during pregnancy are recommended.

## INTRODUCTION

Sleep is an important determinant of maternal and fetal health, and poor sleep quality in pregnant women has adverse consequences^1^. Sleep problems are frequently experienced by women during pregnancy. Large population studies and meta-analyses have reported that 46–76% of pregnant women experience sleep problems^2–4^. Sleep quality impairment affects the physiological, cognitive/behavioral, emotional, and social health of pregnant women. Sleep problems during pregnancy are associated with preterm labor, prolonged labor, increased risk of cesarean delivery, prenatal and postpartum depression, hypertensive disorders in pregnancy, glucose intolerance, and low APGAR score in newborns^5–7^. According to the meta-analysis result of Liu et al. investigating the effect of sleep problems during pregnancy on mother and baby, it was reported that general sleep disorders were significantly associated with pre-eclampsia, gestational hypertension, gestational diabetes, cesarean section, preterm labor, and large for gestational age^8^. The results suggest that poor sleep quality is an important risk factor for adverse maternal and fetal outcomes. Therefore, it is important to evaluate sleep in pregnant women and to perform interventions to improve sleep quality. All factors that cause poor sleep quality in pregnancy should be investigated to provide interventions to improve sleep quality^2,9^.

Considering that the simplest and basic intervention to improve sleep quality is to provide behavioral change^9^, it may be meaningful to investigate the relationship between sleep and healthy life behaviors in pregnancy. In a study conducted by Du et al. to evaluate the effect of maternal sleep quality in early pregnancy on risk factors and pregnancy outcomes, inadequate physical activity, smoking, and a vegetarian diet were found to be associated with poor sleep quality in pregnancy^6^.

We believe that information describing the relationship between healthy life behaviors and sleep quality in pregnant women can guide health education by health professionals to reduce sleep disturbance. However, there are limited studies examining the relationship between sleep quality in pregnancy and pregnancy-specific healthy life behaviors^10^. The present study was conducted to reveal the relationship between healthy life behaviors and sleep quality in pregnant women.

### Study research questions

1. How do healthy lifestyle behaviors during pregnancy affect the sleep quality of pregnant women?
2. What is the relationship between key components of a healthy lifestyle—such as nutrition, physical activity, and stress management—and sleep quality in pregnant women?
3. Is there a significant difference in sleep quality between pregnant women who adopt healthy lifestyle behaviors and those who do not?

## METHODS

### Study design and participants

This study was conducted using a descriptive and correlational research design. It was carried out between March 2023 and March 2024 across Türkiye through an online survey employing a virtual snowball sampling method. Based on an effect size of 0.165, a power of 80%, and a Type I error rate of 0.05, a total of 282 participants were included in the study.

Pregnant women with a body mass index (BMI) below 30 who voluntarily agreed to participate were included in the research. In the initial phase of the study, the purpose of the research was explained in detail, and a consent button was provided to obtain participants’ approval. Those who consented proceeded to the second page, where they were asked to provide their BMI, calculated as body weight in kilograms divided by the square of height in meters (BMI=kg/m^2^). Participants with a BMI of 30 or above were excluded from the study, as they are classified as "obese pregnant women," and obesity is known to be a direct factor affecting sleep quality in the literature. Women with a BMI below 30 were able to proceed to the next page and access the survey questions.

Exclusion criteria included having any psychiatric diagnosis, experiencing a high-risk pregnancy, using antidepressant medication, and being employed in a job that requires night shift work.

### Data collection and measurements

The data were collected online using a structured questionnaire developed by the researchers through Google Forms. Access to the survey was provided via an informative text outlining the study's purpose, scope, and inclusion criteria, which was shared on social media platforms, particularly in groups targeting pregnant women. Pregnant individuals who met the inclusion criteria and volunteered to participate were able to access the survey via the provided link and were included in the study. At the beginning of the questionnaire, an informed consent button was included to obtain participants’ explicit agreement to take part in the study. Participants who clicked the consent button were directed to the remaining questions in the survey, while those who did not provide consent were automatically excluded by the system. All participants were clearly informed that their personal data would be kept confidential and that all responses would be used solely for scientific purposes. The average time to complete the questionnaire ranged from approximately 5–7 minutes. A total of 11 pregnant women who did not complete the questionnaire in full were excluded from the study. Additionally, since no participants met the predefined exclusion criteria, no further exclusions were necessary.

Data were collected by using the "Personal Information Form," "the Healthy Lifestyle Behaviors in Pregnancy Scale (HLBPS)"and "the Pittsburgh Sleep Quality Index (PSQI)" via google forms. The personal information form was prepared by the researchers by reviewing the literature^2,11^. It includes 17 questions designed to define the characteristics of the participants, such as age, education level, and smoking. HLBPS was developed, validated, and made reliable by Yılmaz and Karahan^12^. The HLBPS is a self-reported five-point Likert scale with 29 items across six subscales (pregnancy responsibility, hygiene, nutrition, physical activity, travel, and acceptance of pregnancy). Items are scored from 5 ("always") to 1 ("never"). The scale ranges from 29 to 145, and high scores indicate that healthy living behaviors are positive/high. Cronbach's alpha for the total scale was 0.83^12^, and for this study, 0.83.

The PSQI was developed by Buysse et al.^13^ and adapted into Turkish by Agargun^14^. The PSQI is a widely used tool in studies. The PSQI is a self-report scale that evaluates sleep quality and sleep disturbance in the past 1 month. The scale includes 24 questions: 19 self-reported and 5 for a spouse or roommate. Eighteen scored items span seven components: Subjective Sleep Quality, Sleep Latency, Sleep Duration, Habitual Sleep Efficacy, Sleep Disorders, Sleep Medication Use, and Daytime Dysfunction, each rated 0–3. The component scores sum to a total score from 0 to 21, with scores above five indicating "poor sleep quality." PSQI's overall score ranges from no to severe sleep difficulty, with diagnostic sensitivity of 89.6% and specificity of 86.5%. Cronbach's alpha reliability is 0.88 for the scale^14^ and 0.84 in this study.

### Data analysis

The data obtained in the study were analyzed with IBM SPSS V23 and IBM AMOS V24. Descriptive characteristics of the pregnant women who participated in the study were given as frequency, percentage, mean, and standard deviation. Compliance with normal distribution was examined by multiple normality assumptions and Kolmogorov-Smirnov tests. PATH analysis was used to test the model, and the ML (maximum likelihood) method was used as the calculation method since the assumption of multiple normality was met in testing the structural model. Significance level was taken as p<0.050.

### Ethical considerations

Ethics committee permission was obtained from the "University of Health Sciences" non-interventional research ethics committee. Before sending the data form to the participants, detailed information about the study was given, and the data form was sent to women who volunteered to participate in the study. Informed consent was obtained from the women participating in the study. Thus, the Declaration of Helsinki Principles and Publication Ethics were followed at all stages of the study.

## RESULTS

The mean age of participants was 27.43±5.54 years. Most were housewives (75.9%), married (98.6%), had income equal to expenses (61.7%), and 34.8% had a high school education. The mean gestational week was 24.32±10.4, ranging from 5 to 40 weeks. 18% of pregnant women were in the first trimester, 35.5% in the second trimester, and 46.5% in the third trimester. The mean scores for the HLBPS were as follows: pregnancy responsibility 17.84±3.42, hygiene 17.75±2.99, nutrition 29.85±6.09, physical activity 9.60±3.17, travel 18.72±5.49, pregnancy acceptance 15.85±3.40, and the total HLBPS score 109.62±16.46. The mean PSQI score was 6.90±3.37, and 44.3% of pregnant women had a PSQI ≥5. A statistically significant relationship was not found between PSQI and total and sub-dimension scores of the HLBPS (p>0.050) (Table 1).

**Table 1:** Descriptive statistics of the healthy lifestyle behaviors during pregnancy scale and the Pittsburgh Sleep Quality Index, and the relationship between the scales.

	X	SD	M	Min.	Max.	r	p
Pregnancy responsibility	17.84	3.42	20.00	4.00	20.00	0.021	0.723
Hygiene	17.75	2.99	19.00	4.00	20.00	-0.081	0.173
Nutrition	29.85	6.09	30.00	9.00	45.00	-0.068	0.253
Physical activity	9.60	3.17	9.00	3.00	15.00	-0.039	0.514
Travel	18.72	5.49	20.00	5.00	25.00	-0.041	0.495
Accepting pregnancy	15.85	3.40	16.00	4.00	20.00	-0.077	0.199
HLBPS total score	109.62	16.46	111.00	29.00	14.00	-0.083	0.165
PSQI total score	6.90	3.37	6.00	0.00	18.00		

X: mean; SD: standard deviation; M: median; Min.: minimum; Max.: maximum; HLBPS: Healthy Lifestyle Behaviors in Pregnancy Scale; PSQI: Pittsburgh Sleep Quality Index; r: Spearman's rho correlation coefficient; p<0.05.

**Figure 1:**
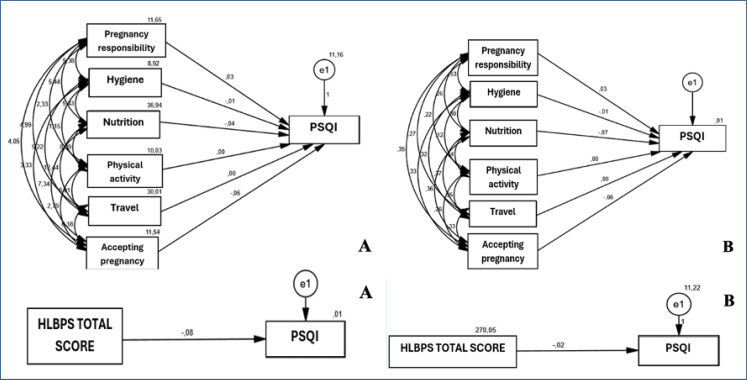
Unstandardized (A) and standardized (B) beta coefficients (for sub-dimensions and total score).

Sub-dimensions of the HLBPS did not have a statistically significant effect on PSQI (p>0.050). There was no statistically significant effect of the total score of the HLBPS on PSQI (p>0.050) (Table 2) (Figure 1).

**Table 2:** Investigation of the effect of healthy lifestyle behaviors in pregnancy scale and sub-dimension scores on Pittsburgh Sleep Quality Index.

Dependent variable		Independent variable	β1	β2	SE	Test statistics	p	r^2^
PSQI	<---	Pregnancy responsibility	0.029	0.029	0.071	0.403	0.687	0.012
PSQI	<---	Hygiene	-0.005	-0.006	0.082	-0.069	0.945	
PSQI	<---	Nutrition	-0.073	-0.040	0.039	-1.023	0.306	
PSQI	<---	Physical activity	-0.003	-0.003	0.073	-0.038	0.970	
PSQI	<---	Travel	0.001	0.001	0.042	0.015	0.988	
PSQI	<---	Accepting pregnancy	-0.064	-0.063	0.067	-0.942	0.346	
PSQI	<---	HLBPS total score	-0.082	-0.017	0.012	-1.378	0.168	0.007

β1: standardized beta coefficient; β2: unstandardized beta coefficient; SE: standard error; r^2^: coefficient of determination; PSQI: Pittsburgh Sleep Quality Index; HLBPS: Healthy Lifestyle Behaviors in Pregnancy Scale; p<0.05.

## DISCUSSION

During pregnancy, improving sleep quality is important for a healthy pregnancy outcome. In this study, the mean score of the "Pittsburgh Sleep Quality Index" of pregnant women was 6.90±3.37, indicating poor sleep quality. In addition, consistent with previous studies, we found that 44.3% of pregnant women had poor sleep quality. Our findings are consistent with previous studies showing that poor sleep quality is common during pregnancy and that impaired sleep during pregnancy may be an important intervention target, as it predicts depression and impaired postpartum sleep^2,4,7^.

In the present study, the relationship between healthy life behaviors during pregnancy and sleep quality was investigated, and the PATH analysis results showed that there was no statistically significant relationship between sleep quality and total and subscale scores of the healthy life behaviors during pregnancy scale.

There are limited studies examining the relationship between sleep quality in pregnancy and a healthy lifestyle. There are studies showing that caffeine intake, smoking, low physical activity, and nutritional characteristics negatively affect sleep quality in pregnant women and that interventions for them improve sleep quality^6,15^. Du et al. reported that insufficient physical activity, smoking, and a vegetarian diet were associated with poor sleep quality in pregnancy^6^. In the literature, there are systematic reviews and meta-analyses showing that moderate- or low-intensity exercise or physical activity interventions improve sleep quality in pregnant women^16–18^. However, there is a limited number of studies in the literature examining the relationship between sedentary life in pregnancy and sleep quality. Consistent with our study findings, Whitaker et al. investigated the association of sleep with sedentary behavior and physical activity patterns during pregnancy and found no association between sedentary behavior and sleep quality^19^. In the literature, there are various studies showing that Mediterranean-type nutrition and physical activity positively affect sleep quality^20,21^. In a cohort study examining sleep and dietary patterns in pregnancy (26–28 weeks, n=497), better sleep quality was observed in those who ate a vegetable-fruit-rice diet compared to those who ate seafood-noodles, but no link was found between short sleep and diet^22^. The nutrition subscale of the HLBPS scale used in this study does not assess the type of diet or types of food consumed per day^11^.

To our knowledge, this is the first study to investigate the relationship between pregnancy-specific healthy life behaviors and sleep quality. The strength of our study is that this relationship was analyzed by PATH analysis. The results show that pregnancy-specific healthy life behaviors do not affect the sleep quality of pregnant women. However, our study has some limitations. Sleep during pregnancy is affected by many factors that are not easy to control such as hormones and physiologic changes as well as social, mental and physical factors. The strongest independent risk factor for poor sleep quality in pregnant women is pregnancy complaints^2,3^. The most important limitation of our study is that these complaints were not questioned. Another important limitation of this study is that psychosocial factors affecting sleep were not investigated. In addition, considering that sleep during pregnancy is a dynamic process, sleep quality was determined by a single assessment in our study and our results include recall bias because our data collection tools were self-report scales. Therefore, we suggest that the relationship between healthy life behaviors and sleep quality should be investigated in different samples and longitudinally, considering pregnancy complaints. In this way, more information can be obtained about the longitudinal relationship between sleep during pregnancy and objectively measured lifestyle behaviors.

## CONCLUSION

Pregnant women often experience sleep disturbances, which can negatively impact maternal and fetal health. This study examined the relationship between sleep quality and key healthy lifestyle behaviors—nutrition, physical activity, and stress management using PATH analysis. The findings revealed no statistically significant association between these behaviors and sleep quality during pregnancy. Additionally, no significant difference in sleep quality was observed between pregnant women who adopted healthy lifestyle behaviors and those who did not. Based on these results, we recommend future longitudinal studies with diverse samples to further investigate the potential links between healthy lifestyle behaviors and sleep quality during pregnancy.

## Data Availability

The datasets generated and/or analyzed during the current study are available from the corresponding author upon reasonable request.
